# The accuracy of three-dimensional rapid prototyped surgical template guided anterior segmental osteotomy

**DOI:** 10.4317/medoral.23009

**Published:** 2019-08-19

**Authors:** Moyuan Qu, Songsong Zhu, Zhiai Hu, Yunfeng Li, Bassam Abotaleb, Ruiye Bi, Nan Jiang

**Affiliations:** 1 DDS. State Key Laboratory of Oral Diseases, National Clinical Research Center for Oral Diseases, West China Hospital of Stomatology, Sichuan University, Chengdu, China; 2 PhD, MD. State Key Laboratory of Oral Diseases, National Clinical Research Center for Oral Diseases, West China Hospital of Stomatology, Sichuan University, Chengdu, China; 3 DDS, PhD. State Key Laboratory of Oral Diseases, National Clinical Research Center for Oral Diseases, West China Hospital of Stomatology, Sichuan University, Chengdu, China

## Abstract

**Background:**

Surgical guiding templates provided a reliable way to transfer the simulation to the actual operation. However, there was no template designed for anterior segmental osteotomy so far. The study aimed to introduce and evaluate a set of 3D rapid prototyping surgical templates used in anterior segmental osteotomy.

**Material and Methods:**

From August 2015 to August 2017, 17 patients with bimaxillary protrusions were recruited and occlusal-based multi-sectional templates were applied in the surgeries. The cephalometric analysis and 3D superimposition were performed to evaluate the differences between the simulations and actual post-operative outcomes. The patients were followed-up for 12 months to evaluate the incidence rate of complications and relapse.

**Results:**

Bimaxillary protrusion was corrected in all patients with no complication. In radiographic evaluations, there was no statistically significant difference between the actual operations and the computer-aided 3D simulations (*p* >0.05, the mean linear and angular differences were less than 1.32mm and 1.72° consequently, and 3D superimposition difference was less than 1.4mm). The Pearson intraclass correlation coefficient reliabilities were high (0.897), and the correlations were highly significant (*P*< 0.001).

**Conclusions:**

The 3D printed surgical template designed in this study can safely and accurately transfer the computer-aided 3D simulation into real practice.

** Key words:**CAD/CAM; anterior segmental osteotomy; surgical guiding templates; bimaxillary protrusion; virtual surgery simulation.

## Introduction

Bimaxillary protrusion (BP) is a common type of dento-maxillofacial deformities, predominantly in African and Asian adults (1,2) The main clinical manifestations of BP are protrusive and proclined incisors, which are usually combined with lip incompetence, gummy smile, mentalis strain, receding chin, and anterior open bite.

Orthodontic treatment and orthognathic surgical treatment are the treatment strategies of BP. The orthodontic treatments can make a good effect for patients with proclined incisors and coordinated maxilla and mandible, while orthognathic surgeries are usually used to the patients with relatively normal incisors along with horizontal prognathic maxilla and mandible (3,4). In the BP patients with Angle Class I malocclusion, the general surgical approach is anterior segmental osteotomy(ASO) which is also useful for correcting dental spaces, anterior open bite, and other dento-maxillofacial deformities (5,6). Compared with other orthognathic surgeries, ASO has the advantages of the open surgical field, stable occlusal molar relation, almost no impact on the temporomandibular joint, and low rate of relapse. However, some studies reported that ASO related complications include accidental fractures, canine pulp devitalization, periodontal bone losses, tooth losses, and unintended nasal changes (7-10).

With the rapid development and application of Virtual surgical planning and rapid prototyping (RP) technology, the three-dimensional (3D) technique can provide new possibilities for the treatment of complex dento-maxillofacial deformities and simulate the process and effects of the orthognathic surgeries (11-15). Compared with the traditional surgical planning, the computer-aided surgical simulation has many advantages, which include: 1) comprehensive 3D evaluation and diagnosis. 2) predictable change of hard and soft tissue. 3) simulation of different surgical procedures to obtain the best possible outcomes (13). These goals can be achieved through the design and manufacture of 3D-printing surgical templates and the production of cutting guides can allow the surgeon to intraoperatively reproduce the virtually planned osteotomy and refixation (16-18).

Nevertheless, there was no template designed for ASO so far. The main purpose of this study is to introduce a set of templates transferring the virtual surgical simulation of ASO to the real operation room. This set of templates can guide both the osteotomy and reposition the osteotomized parts with the advantage of simplified preoperative and operative procedures with no need for the traditional model and the additional preparation as well as avoiding the associated errors. Cephalometric analysis and 3D superimposition were performed to compare the differences between the simulated and actual post-operative outcomes.

## Material and Methods

-Patients selection

To explore the effect of the templates designed for ASO, 17 consecutive healthy adult patients with the typical facial bimaxillary protrusion along with molar Angle Class I occlusions were enrolled in this study at the Center of Orthognathic Surgery, West China Hospital of Stomatology, Sichuan University (Chengdu, China) between August of 2015 to August of 2017. The study protocol was approved by the West China Hospital of Stomatology Institutional Review Board, and all participants signed the informed consent form.

-Virtual surgery simulation and templates design

All patients underwent radiographic examinations (panoramic X-ray, lateral and frontal cephalogram, and spiral CT). The three-dimensional models were reconstructed using a high-resolution spiral computed tomography (CT) and the surface scanning of maxillary and mandibular impressions generated by a laser surface scanner (3 shapes, Copenhagen, Denmark). The spiral CT and skeletal images were imported into Dolphin Imaging 11.7 Premium (Dolphin Imaging and Management Solutions, Chatsworth, CA) and Mimics software (version 10.01; Materialise, Leuven, Belgium) to perform the Surgical planning, simulated osteotomies and repositioned bony segments as it is in our previous studies (19).

**Figure 1:**
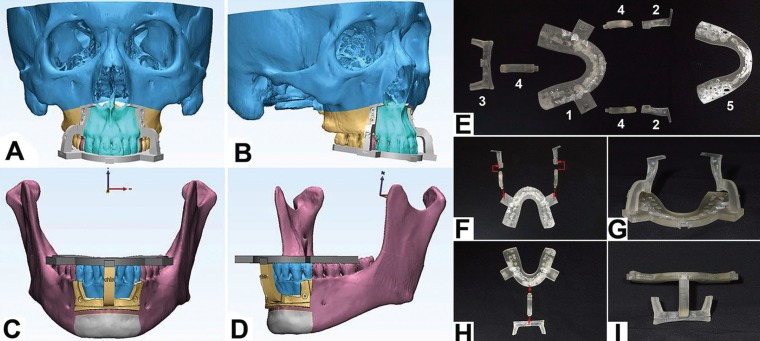
The design and the composition of the templates. A-D: The simulated osteotomies and the design of surgical guiding templates in the surgical planning software; E-I: Photos of the surgical guiding templates; E: Components of a whole system of surgical guiding templates. 1 and 5: Two occlusal splints, the most important part in the whole system of guiding templates. Occlusal relations of preoperation and simulated postoperation were imprinted separately on the intermediate occlusal splint (1) and the final occlusal splint (5). 2 and 3: Three bone attachments, covering the area of osteotomy to guide the osteotomy line; 4: Arms connecting the bone attachments with the intermediate occlusal splint; F: The diagram showing the installation of maxillary templates. G: A pair of surgical guiding templates assembled for the maxillary osteotomy; H: The diagram showing the installation of mandibular templates. I, A pair of surgical guiding templates assembled for the mandibular osteotomy.

The design idea is to make the maxillary molars as reference landmarks to retain the original occlusal relation, guide the position and direction of the osteotomies and positioning of anterior segments (Fig. 1). The intermediate occlusal splint was designed and fabricated to reset and support the original stable occlusion and help the other parts of the guiding templates to be fixed in the accurate place as the simulation. Bone attachments covering the redundant bones can guide the osteotomies. Furthermore, the final occlusal splint was designed to maintain the original stable occlusion of the molar teeth, guide the final locations of anterior bony segments and be used for intermaxillary fixation.

The surgical guiding templates were fabricated from polypropylene by rapid prototyping and subjected to low-temperature sterilization.

-Surgical procedures with guiding templates

**Figure 2:**
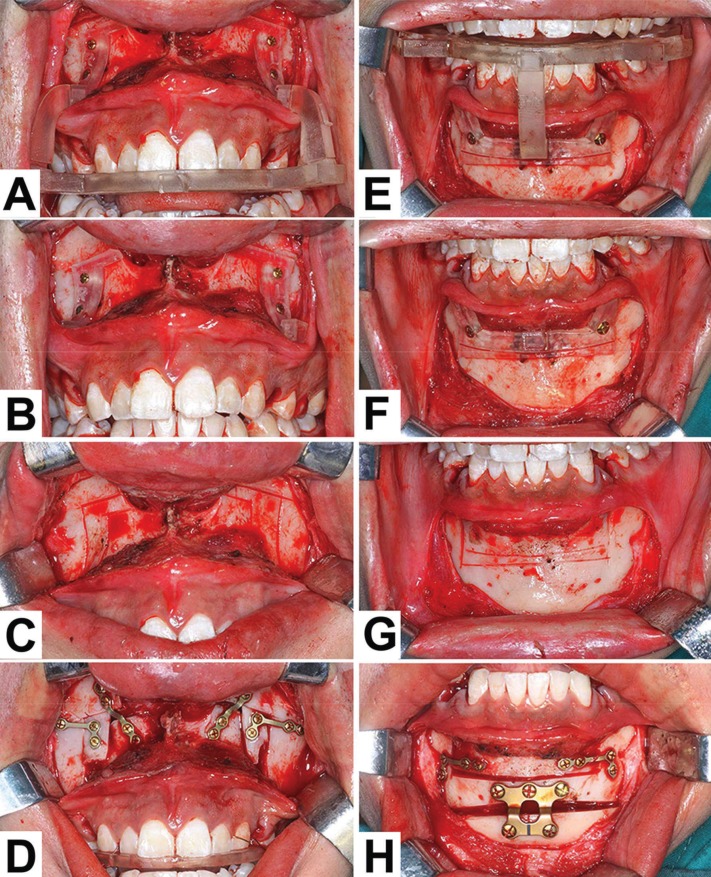
The application of the surgical templates in ASO. A, E, Placement and fixation of the bone attachments with the guidance of the intermediate occlusal splint and arms. B, F, After the extraction of the intermediate occlusal splint and arms, the bone attachments can guide the osteotomies. C, G, Osteotomy lines with the guidance of the bone attachments. D, H, The final locations and fixations of anterior segments.

The surgeries were performed under general anesthesia. After the extractions of the premolars, the operation was carried out according to Cupar and Hullihen procedures (5,20). According to the position of the intermediate splint, the bone attachments were connected, located and fixed like the simulation. (Fig.2 A,E). And then, the surgical osteotomy procedures are shown in the (Fig.2 A-G).

In the cases where Orthodontic brackets were not used, four skeletal anchorage screws were implanted for elastic traction. Eventually, the anterior bones were fixed with titanium plates and screws. Genioplasty was done if needed according to preoperative planning and simulation.

-Comparison of Simulated and Actual Postoperative Outcome

The patients were followed up for 12 months and underwent radiographic and clinical examination at the interval of one, three, six and twelve months after the surgeries to objectively evaluate the outcomes and complications. The assessing variables of complications included; teeth damage, pulp vitality, nonunion, malocclusion, infection, numbness, and the negative facial changes.

To evaluate the accuracy of the operative procedure with the assistant of the templates, we selected the radiographs of one-month post operation (panoramic X-ray, lateral cephalogram, frontal cephalogram, and spiral CT) for cephalometric analysis. SN plane, Frankfort horizontal plane and Bolton plane are relatively stable planes in the cephalometric analysis. Measuring the distances between landmarks and the three planes is valuable to multi-evaluate the movements of the landmarks (16). After ASO, the positions of the anterior landmarks moved with the anterior bony segments. So, we selected 4 cephalometric points and 4 angles to evaluate the differences between the simulated and actual post-operative outcomes. Moreover, 3D superimposition of the virtual and actual results had been done.

-Statistical analysis

All statistical data analyses were performed with SPSS version 22.0 (SPSS, Chicago, IL, USA). The reliabilities were qualified using Pearson’s intraclass correlation coefficient (ICC). The differences between 3D virtual surgical simulations and actual surgical outcomes were analyzed using the paired samples t-test. Statistical significance was set at *P* <0.05.

## Results

ASO was performed on all the 17 patients (age between 18-30 years), of which 11 patients were combined with the advancement Genioplasty (chin hypoplasia or airway stenosis).

Following the preoperative 3D simulations, the surgeries of all patients were performed successfully with 3D rapid prototyped surgical templates. The mean amount of surgical movement was 5.33mm for maxilla and 4.75mm for mandible. All patients successfully gained dramatic improvements in their facial appearance and expressed satisfaction with the outcomes. None of the patients suffered relapse or complications like damage to teeth, pulp vitality, nonunion, malocclusion, infection, persistent numbness, negative facial change.

The results of linear and angular measurements for all patients were shown in  Table 1 and  Table 2. The Pearson intraclass correlation coefficient reliabilities were high (0.897), and the correlations were highly significant (*P*< 0.001). The paired samples t-tests were adopted for the comparisons between virtual and actual surgical outcomes and the results showed no statistical significance in all comparisons (*p*>0.05). Moreover, the mean linear and angular differences between actual and virtual surgical outcomes were less than 1.32mm and 1.72°.

**Table 1:**
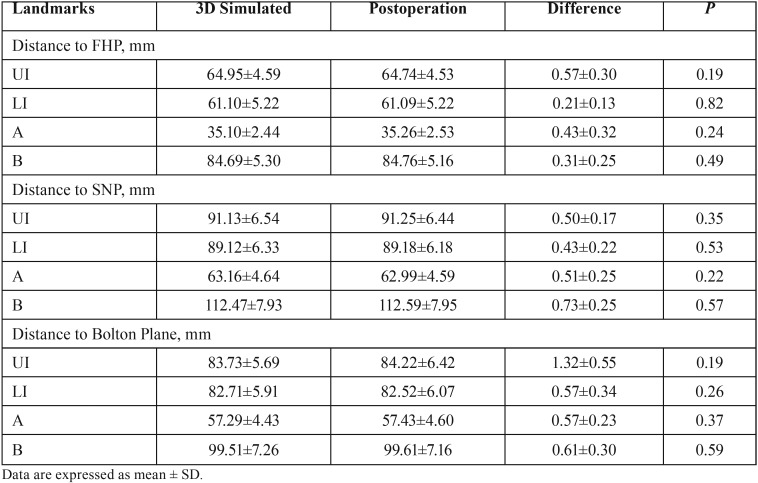
The results of linear measurements for all patients.

**Table 2:**
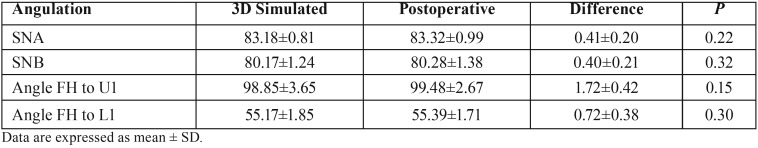
The results of angular measurements for all patients.

**Figure 3:**
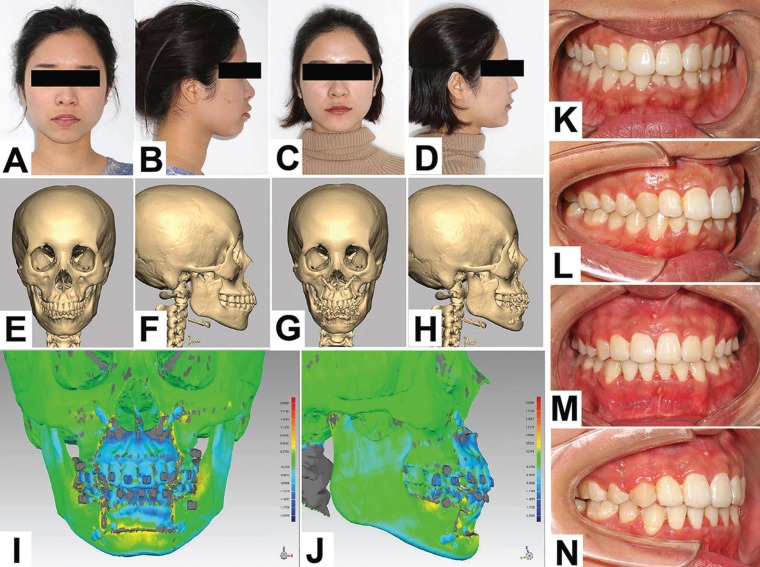
The case pictures of a representative patient who complained of an unfavorable face type for ten years. A-D: The facial profiles before (A, B) and after (C, D) the template guided ASO; E-H: The 3D reconstructed spiral CT images before (E, F) and after (G, H) the template guided ASO; I and J: The superimposed 3D images of virtual and actual outcomes of the representative patient; K-N: The contrast of preoperative (K, L) and postoperative (M, N) occlusion of the representative patient.

A 23 years old patient was chosen as a representative case to illustrate the changes in the facial profile, occlusal relation, and radiographs (Fig. 3). The results of the superimposition difference between the virtual and actual results were between -0.8 and 1.4 mm (Fig. 3, I and J).

## Discussion

Anterior segmental osteotomy (ASO) is one of the most frequently-used operation in orthognathic surgery. However, studies reported that ASO related complications include accidental fractures, canine pulp devitalization, periodontal bone losses, tooth losses, and unintended nasal changes. Therefore, a more effective technique that can improve the safety and accuracy of the ASO is required.

The computer-aided design could be useful for such purpose with increased precision and reduced morbidity and operative time. Several studies indicated that guiding templates made by three-dimensional rapid prototyping technique could allow the surgeon to intraoperatively reproduce the virtually planned osteotomy and reposition. Zinser *et al.* reported that a 3D-surgical splint technique provided a reliable method to transfer the simulation to the actual operation(16). Polley *et al.* designed an orthognathic positioning system to guild the Le Fort I osteotomy, sagittal split ramus osteotomy, and genioplasty (17). Moreover, Ye *et al.* applied the computer image-guided template for mandibular angle osteotomy (18). And in this study, to facilitate the procedure with improvement in the accuracy and safety of ASO, a set of occlusal-based multi-sectional templates had been designed.

In all cases rerolled in this study, the mean of linear and angular differences between actual and virtual surgical outcomes were less than 1.32mm and 1.72° (*p*>0.05). These findings were relatively constant with the previous studies. Zinser *et al.* showed the mean linear and angular differences of less than 0.75mm and 0.94° with the assistance of the templates (16). Moreover, Li *et al.* measured a difference of less than 1.1mm (21). Previous studies urged that if positional differences between actual and virtual surgical outcomes were less than 2 mm and orientation differences were less than 4°, then the orientation differences could be considered clinically inconsequential (22,23). Consequently, the ASO with the guidance of the 3D-printing templates designed in this study can be considered as an accurate surgical procedure.

In orthognathic surgery, surgeons were very sensitive about the stable position of condyles because it can prevent the TMJ symptom and the recurrence of malocclusion after the operations (24). In our study, the operations were performed based on retaining the original occlusal relation. Moreover, the final occlusal splints prevented the supracontact of anterior teeth, so the condyles could be kept stable in the original position. And during the postoperative follow-up, there were no TMJ symptom occurred.

Even with development and improvement of computer-aided design and 3D-printing surgical template, challenges remain in terms of accurately simulating the changes of soft tissue, so the facial profiles are hard to be absolutely the same with the surgical simulations after operations (25-27). In this study, we just introduced and demonstrated the safety and accuracy of the template guided ASO. Next step, we plan to design a randomized controlled trial with more clinical cases and longer follow-up period to compare the safety, accuracy and stability between the template guided ASO and the traditional procedure.
